# Obesity and the relation between joint exposure to ambient air pollutants and incident type 2 diabetes: A cohort study in UK Biobank

**DOI:** 10.1371/journal.pmed.1003767

**Published:** 2021-08-30

**Authors:** Xiang Li, Mengying Wang, Yongze Song, Hao Ma, Tao Zhou, Zhaoxia Liang, Lu Qi

**Affiliations:** 1 Department of Epidemiology, School of Public Health and Tropical Medicine, Tulane University, New Orleans, Louisiana, United States of America; 2 Department of Epidemiology and Biostatistics, School of Public Health, Peking University, Beijing, China; 3 School of Design and the Built Environment, Curtin University, Bentley, Perth, Western Australia, Australia; 4 Department of Epidemiology and Biostatistics, School of Public Health (Shenzhen), Sun Yat-sen University, Guangzhou, Guangdong, China; 5 Department of Obstetrics, Women’s Hospital, Zhejiang University School of Medicine, Hangzhou, Zhejiang Province, China; 6 Department of Nutrition, Harvard T.H. Chan School of Public Health, Boston, Massachusetts, United States of America; Monash University, AUSTRALIA

## Abstract

**Background:**

Air pollution has been related to incidence of type 2 diabetes (T2D). We assessed the joint association of various air pollutants with the risk of T2D and examined potential modification by obesity status and genetic susceptibility on the relationship.

**Methods and findings:**

A total of 449,006 participants from UK Biobank free of T2D at baseline were included. Of all the study population, 90.9% were white and 45.7% were male. The participants had a mean age of 56.6 (SD 8.1) years old and a mean body mass index (BMI) of 27.4 (SD 4.8) kg/m^2^. Ambient air pollutants, including particulate matter (PM) with diameters ≤2.5 μm (PM_2.5_), between 2.5 μm and 10 μm (PM_2.5–10_), nitrogen dioxide (NO_2_), and nitric oxide (NO) were measured. An air pollution score was created to assess the joint exposure to the 4 air pollutants. During a median of 11 years follow-up, we documented 18,239 incident T2D cases. The air pollution score was significantly associated with a higher risk of T2D. Compared to the lowest quintile of air pollution score, the hazard ratio (HR) (95% confidence interval [CI]) for T2D was 1.05 (0.99 to 1.10, *p* = 0.11), 1.06 (1.00 to 1.11, *p* = 0.051), 1.09 (1.03 to 1.15, *p* = 0.002), and 1.12 (1.06 to 1.19, *p* < 0.001) for the second to fifth quintile, respectively, after adjustment for sociodemographic characteristics, lifestyle factors, genetic factors, and other covariates. In addition, we found a significant interaction between the air pollution score and obesity status on the risk of T2D (*p*-interaction < 0.001). The observed association was more pronounced among overweight and obese participants than in the normal-weight people. Genetic risk score (GRS) for T2D or obesity did not modify the relationship between air pollution and risk of T2D. Key study limitations include unavailable data on other potential T2D-related air pollutants and single-time measurement on air pollutants.

**Conclusions:**

We found that various air pollutants PM_2.5_, PM_2.5–10_, NO_2,_ and NO, individually or jointly, were associated with an increased risk of T2D in the population. The stratified analyses indicate that such associations were more strongly associated with T2D risk among those with higher adiposity.

## Abstract 2

Xiang Li and co-workers study the potential influence of obesity on associations between air pollutants and incidence of type 2 diabetes.

## Author summary

**Why was this study done?:**

- Recent evidence has linked ambient air pollution with type 2 diabetes (T2D). However, most of them were mainly focused on 1 or 2 air pollutants separately. In addition, it has been reported that the association between air pollution and T2D was augmented among people with increased adiposity.
- Individuals are usually exposed to various air pollutants simultaneously; and the importance of assessing multi-air pollutant exposures as a whole has been increasingly recognized.
- Thus, in this study, we aimed to analyze the association between the air pollution score that incorporates various air pollutants with the risk of T2D. We also particularly examined the potential modification by obesity.

**What did the researchers do and find?:**

- We built an air pollution score, incorporating PM_2.5_, PM_2.5–10_, nitrogen dioxide (NO_2_), and nitric oxide (NO), and analyzed its association with the risk of T2D among 449,006 participants from UK Biobank. Air pollutants were measured in 2010. During a median follow-up time of 11 years, 18,239 T2D cases were documented.
- We found that long-term exposure to various air pollutants, individually or jointly, was associated with an increased risk of T2D. Moreover, such association was more pronounced among people with increased adiposity.

**What do these findings mean?:**

- Our findings highlight the importance of comprehensively assessing various air pollutants as a whole in the prevention of T2D.
- Given the adverse impact of air pollution on T2D, attention should be paid to reducing and mitigating air pollution.
- Our results also emphasize the merit of obesity management.

## Introduction

Type 2 diabetes (T2D) is a persistent public threat worldwide. The prevalence of T2D has been increasing, and it is set to rise even further. From the most recent International Diabetes Federation (IDF), it is estimated that there will be more than 700 million adults living with diabetes worldwide by 2045 [1]. While several traditional risk factors, such as poor diet [2,3], low physical activity [4,5], and poor sleep behaviors have been related to T2D [6,7], recent evidence suggests that ambient air pollution may also contribute to the development of the disease [8–10]. However, the relations between air pollution and T2D is a relatively new field, and the previous findings between air pollution and T2D were inconsistent [11,12]. Notably, the prior studies are largely limited by cross-sectional design [13–15], and small sample size [16], while data from large-scale, cohort settings are still scarce. In addition, individuals are usually exposed to a combination of various air pollutants simultaneously, and the importance of assessing multi-air pollutant exposures as a whole has been increasingly recognized [17–19]. However, most previous studies were mainly focused on 1 or 2 air pollutants separately, without considering the joint exposure to various air pollutants [15,20–23]. Recently, we have created and validated a novel air pollution score incorporating various air pollutants, which has been shown to be associated with heart failure [24]. To date, no study has jointly investigated long-term exposure to various air pollutants with the risk of T2D in a cohort study design.

Of note, emerging evidence has also linked air pollutants such as PM_2.5_, nitrogen dioxide (NO_2_), and O_3_ with increased adiposity [25,26], and it was reported that the association between air pollutants and T2D was augmented in obese participants [27]. Therefore, we hypothesized that obesity status might modify the relationship between air pollution and the risk of T2D. In addition, prior evidence suggests that the genetic factors may modify the environment–disease relation, while investigations on the modification effect by the genetic predisposition on the relation between air pollution and T2D risk are scarce [28].

In the present study, we aimed to analyze the associations of various air pollutants and the air pollution score, which comprehensively incorporated PM_2.5_, PM_2.5–10_, NO_2,_ and nitric oxide (NO), with the risk of T2D among 449,006 participants from UK Biobank. We particularly examined the potential modification by obesity status (both general and central obesity) and genetic predisposition to obesity or T2D.

## Method

### Protocol

This research has been conducted using the UK Biobank Resource under Application Number 29256. Our study did not employ a prospective protocol. Analyses were first planned and performed in January 2021. During the peer review, we added a table of Pearson correlations between each of the air pollutant and a map of air pollution levels exposed to study participants at baseline (2010). Minor changes to the manuscript were also made at the request of peer reviewers.

### Study population

The UK Biobank is a prospective cohort based in the United Kingdom aimed to improve the prevention, diagnosis, and treatment of a wide range of illnesses. Briefly, over 500,000 middle-aged participants were recruited across 2006 to 2010. Participants provided a wide range of health-related information through touchscreen questionnaires, physical measurements, and biological samples at baseline or follow-up assessment. Details of the study design have been described elsewhere previously [29]. The study was approved by both the National Health Service National Research Ethics Service (Ref: 11/NW/0382) and the Institutional Review Board of Tulane University (2018–1872). All the participants provided written informed consent.

In the current analysis, we excluded participants with T2D (*N =* 12,185) and those with missing information on residential air pollution (*N* = 41,302) at baseline, leaving a total of 449,006 participants for the primary analysis. In the joint association of genetic risk and air pollution analysis, to avoid heterogeneity, we only included participants of European descent with complete genotyping data (*N* = 417,035).

### Assessment of air pollution and air pollution score

The annual average air pollution, including PM_2.5_, PM_2.5–10_, NO_2_, and NO_x_, in 2010, were modeled for each address using a Land Use Regression (LUR) model developed as part of the European Study of Cohorts for Air Pollution Effects (ESCAPE, http://www.escapeproject.eu/) [30,31] and linked to participants’ residential addresses given at baseline visit. The LUR models calculated the spatial variations of annual average air pollutant concentration at participants’ home addresses using Geographic Information System (GIS)-derived predictors, such as traffic, land use, and topography. The LUR model is based on ESCAPE monitoring done between January 2010 and January 2011, and air pollution estimates are representative for the year 2010. The ESCAPE estimates for particulates are valid up to 400 km from the monitoring area [30,31]. The concentration of NO is estimated by subtracting NO_2_ from NO_x_ following the previous study [32].

To capture the joint exposure to various air pollutants, we created an air pollution score by summing up concentrations of 4 ambient air pollutants (PM_2.5_, PM_2.5–10_, NO_2_, and estimated NO), weighted by the multivariable-adjusted risk estimates (β coefficients) on T2D in the present analysis [24]. The β coefficient was from the final model with individual air pollutant as the independent variable, one at a time. The equation was: air pollution score = (β_PM2.5_ × PM_2.5_ + β_PM2.5–10_ × PM_2.5–10_ + β_NO2_ × NO_2_ + β_NO_ × NO) × (4 / sum of the β coefficients). The air pollution score ranges from 31.7 to 140.3, a higher score indicating greater exposure to ambient air pollution. Since PM_2.5–10_ was not significantly associated with T2D, we also created a weighted air pollution score without PM_2.5–10_.

### Assessment of outcomes

Information on incident T2D was collected through February 8, 2020. Incident T2D was defined by ICD-10 code E11, ascertained by hospital inpatient records containing data on admissions and diagnoses from the Hospital Episode Statistics for England, Scottish Morbidity Record data for Scotland, and the Patients Episode Database for Wales.

### Genotype data

Genotyping, imputation, and quality control of the genetic data were performed by the UK Biobank team. The detailed information is available elsewhere (http://www.ukbiobank.ac.uk/scientists-3/genetic-data/) [33]. A genetic risk score (GRS) for T2D was created using 112 independent single nucleotide polymorphisms (SNPs), which passed quality control out of the 128 SNPs recently identified to be associated with T2D at genome-wide significance [34,35]. Information of the 112 independent SNPs is provided in **S1 Table**. The GRS for T2D was calculated by the weighted method: GRS = (β_1_ × SNP_1_ + β_2_ × SNP_2_ + … + β_112_ × SNP_112_) × (112 / sum of the β coefficients). Each SNP was recoded as 0, 1, and 2 according to the number of risk alleles. The β coefficient was obtained from the reported GWAS meta-analysis [35]. The GRS for T2D in the current analysis ranges from 81.0 to 136.9; a higher score indicates a higher genetic predisposition to T2D. The BMI-GRS was created in the same way as T2D-GRS, using the 97 identified body mass index (BMI)-related SNPs [36]. Information of the BMI-related SNPs is summarized in **S2 Table**. We determined whether participants were at low, intermediate, or high genetic risk according to the tertile categories of the GRS.

### Assessment of other covariates

Age, sex, ethnicity, and Townsend deprivation index (based on the participant’s postcode, higher scores indicate a higher degree of deprivation) were obtained at baseline. Weight, height, and waist circumference (WC) were measured at baseline during the initial assessment center visit. BMI was calculated as weight divided by height squared (kg/m^2^) during the initial Assessment Centre visit. Overall obesity status was defined as follows: normal weight: 18.5 to less than 25 kg/m^2^; overweight: 25 to less than 30 kg/m^2^; obese: 30 kg/m^2^ and above. Central obesity was defined using the European cutoff points for WC: ≥94 cm for men and ≥80 cm for women. Physical activity was assessed by the International Physical Activity Questionnaire (IPAQ) and metabolic equivalent task (MET) score was calculated using the IPAQ guideline [37,38]. Alcohol intake was assessed by the Touchscreen questionnaire. A healthy diet score was adapted from the American Heart Association Guidelines and defined as adherence to 4 or 5 components of the following: (1) total fruit intake ≥4.5 pieces/week; (2) total vegetable intake ≥4.5 servings/week (3 tablespoons of vegetable considered as 1 serving); (3) total fish intake ≥2 servings/week; (4) processed meat intake less than twice/week; and (5) red meat intake ≤5 times/week [39].

### Statistical analysis

Follow-up time was calculated from the recruitment date to the first diagnosis of T2D, lost to follow-up, death, or end of the current follow-up, whichever came first. Cox proportional hazard models were used to estimate the hazard ratios (HRs) and 95% confidence intervals (CIs) for incident T2D associated with the individual air pollutant or the air pollution score. The proportional hazards assumption was tested by creating a time-dependent variable, and no violation was found. Cox regression models were adjusted for age, sex, and ethnicity in model 1. Model 2 was further adjusted for Townsend deprivation index, assessment center, alcohol intake, smoking status, physical activity, sedentary hours, and healthy diet score. In model 3, we additionally adjusted for BMI, systolic blood pressure, antihypertension medication use, high cholesterol, and T2D-GRS. We examined the dose–response relationship between the air pollution score and incident T2D using the restricted cubic spline analysis. We particularly tested whether the association between the air pollution score and risk of T2D was modified by obesity status (overall obesity and central obesity) or genetic predisposition to T2D or obesity, by including an interaction term between the air pollution score and the continuous BMI or WC. To disentangle the temporal concerns regarding the relationship between air pollution and T2D, we further conducted a sensitivity analysis by excluding T2D cases that occurred in the first 2 years of follow-up. In addition, we performed the analyses among participants who have been living in the current address for at least 5 years to assess the long-term effect of air pollution on T2D.

Statistical analyses were performed with SAS version 9.4 (SAS Institute, Cary, NC). All *p*-values were two-sided, and *p* < 0.05 was considered statistically significant.

The study was reported as per the Strengthening the Reporting of Observational Studies in Epidemiology (STROBE) guidelines (S1 STROBE checklist).

## Results

**Fig 1:**
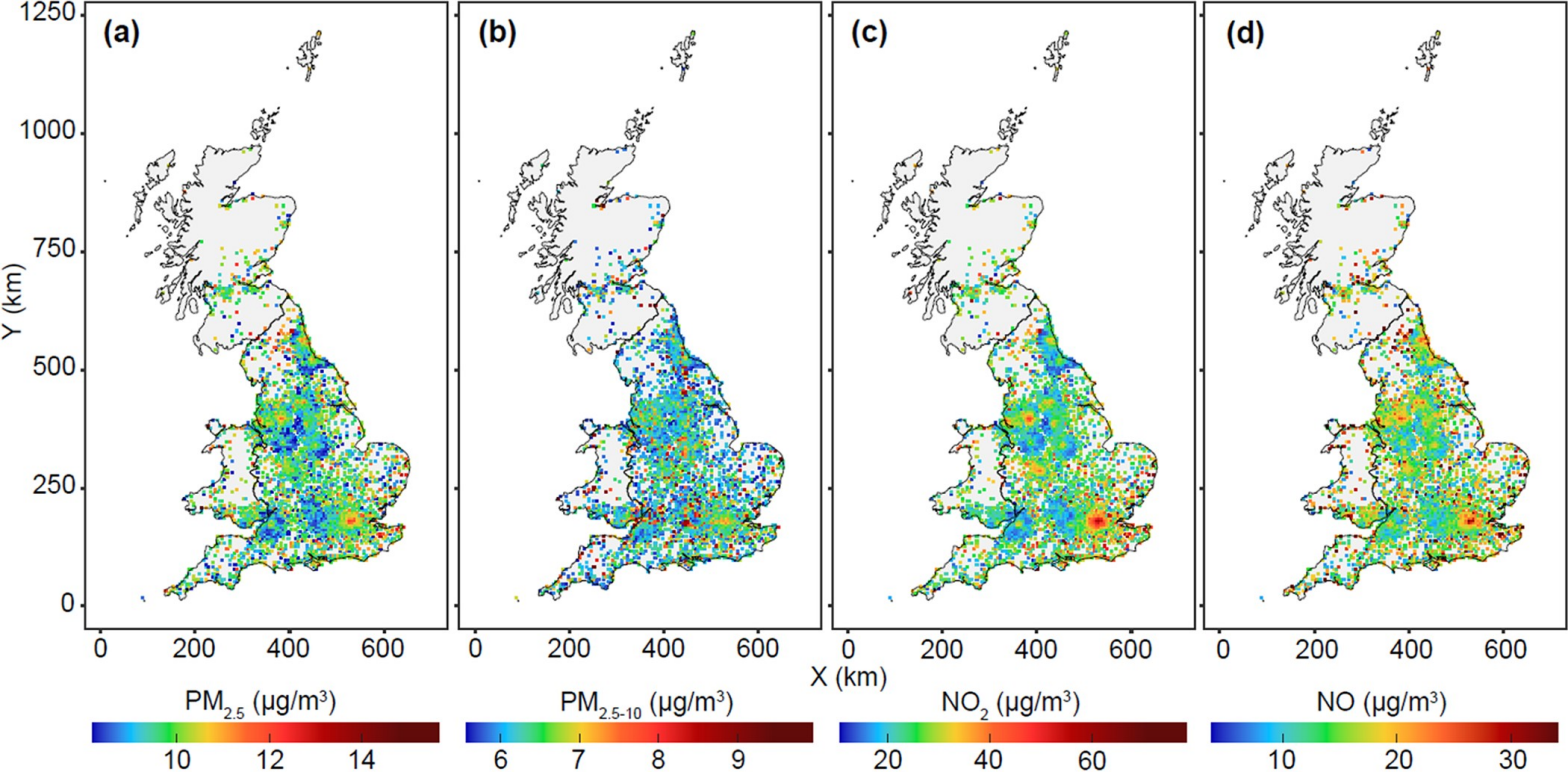
Map of air pollutions (PM_2.5_, PM_2.5–10_, NO_2_, and NO) of areas where participants lived in 2010. (a) PM_2.5_, (b) PM_2.5–10_, (c) NO_2_, and (d) NO. The administrative boundary data of the UK are sourced from UK Government Open Data portal (https://data.gov.uk/dataset/3fd8d2d2-b591-42ff-b333-c53a6a513e96/countries-december-2017-full-clipped-boundaries-in-great-britain). These data are UK government–released open data. NO, nitric oxide; NO_2_, nitrogen dioxide; PM_2.5_, particulate matter with aerodynamic diameter ≤2.5 μm; PM_2.5–10_, particulate matter with an aerodynamic diameter between 2.5 and 10 μm.

Among the 449,006 participants, a total of 18,239 incident cases of T2D were recorded during a median follow-up time of 11 years. Baseline characteristics of the study participants according to the quintiles of the air pollution score are presented in **Table 1**. Participants with higher exposure to ambient air pollution were younger, with a greater Townsend deprivation index (indicating a greater degree of deprivation), more likely to be current smokers, and less likely to be physically active. The Pearson correlation coefficients among the individual air pollutants were shown in **S3 Table**. Air pollution levels in areas where participants lived in 2010 were shown in **Fig 1**. The distribution patterns of air pollution levels in the current study are comparable to the spatial distribution of air pollutants from the public UK Air Information Resources (https://uk-air.defra.gov.uk/data/pcm-data). The administrative boundary data of UK is sourced from UK Government Open Data portal (https://data.gov.uk/dataset/3fd8d2d2-b591-42ff-b333-c53a6a513e96/countries-december-2017-full-clipped-boundaries-in-great-britain). These data are UK government–released open data.

**Table 1:** Baseline characteristics of the UK Biobank participants according to the quintiles of air pollution score (*N =* 449,006).

	Air Pollution Score				
	Q1	Q2	Q3	Q4	Q5
Age, year	57.2 (7.8)	56.9 (8.0)	56.7 (8.1)	56.1 (8.2)	55.4 (8.3)
White	85,814 (95.2)	83,849 (93.6)	82,394 (92.2)	79,749 (89.7)	74,540 (84.1)
Male	40,524 (45.1)	40,443 (45.0)	40,483 (45.1)	40,514 (45.1)	41,078 (45.7)
PM_2.5_, μg/m^3^	8.7 (0.4)	9.5 (0.3)	9.9 (0.3)	10.4 (0.4)	11.5 (0.9)
PM_2.5–10_, μg/m^3^	6.2 (0.8)	6.2 (0.8)	6.3 (0.8)	6.5 (0.9)	6.9 (1.0)
NO_2_, μg/m^3^	17.3 (2.8)	22.8 (2.7)	26.3 (2.8)	29.8 (2.9)	37.0 (6.4)
NO, μg/m^3^	9.4 (2.8)	13.4 (3.6)	16.0 (3.7)	18.9 (3.9)	29.1 (11.9)
BMI, kg/m^2^	27.0 (4.4)	27.3 (4.6)	27.4 (4.7)	27.5 (4.8)	27.5 (5.0)
WC, cm	89.2 (13.0)	89.9 (13.1)	90.3 (13.3)	90.5 (13.4)	90.4 (13.6)
Systolic blood pressure, mm Hg	138.8 (18.5)	138.2 (18.5)	138.1 (18.4)	137.4 (18.4)	136.2 (18.3)
Townsend deprivation index	−3.2 [−4.2–−2.1]	−2.9 [−4.1–−1.3]	−2.4 [−3.7–−0.30]	−1.4 [−3.0–1.0]	0.9 [−1.6–3.6]
Total MET	2,466.0 [1,048.5–3,155.0]	2,435 [996.0–2,979.0]	2,493.0 [1,010.0–2,946.0]	2,559.0 [1,022.0–2,932.0]	2,506.5 [1,032.0–2,882.5]
Healthy diet score	2.1 (0.9)	2.1 (0.9)	2.1 (0.9)	2.1 (0.9)	2.1 (0.9)
Sedentary time	4.0 [3.0–6.0]	4.0 [3.0–6.0]	4.0 [3.0–6.0]	4.0 [3.0–6.0]	4.0 [3.0–6.0]
Smoking status					
Never	52,165 (58.3)	50,860 (56.9)	49,428 (55.4)	48,503 (54.4)	44,740 (50.2)
Previous	30,835 (34.5)	30,926 (34.6)	30,944 (34.7)	30,458 (34.1)	31,140 (35.0)
Current	6,501 (7.3)	7,597 (8.5)	8,902 (10.0)	10,251 (11.5)	13,200 (14.8)
Alcohol intake					
Daily or almost daily	22,284 (24.8)	18,132 (20.2)	17,114 (19.1)	16,624 (18.6)	18,330 (20.5)
3–4 times/week	23,211 (25.9)	21,628 (24.1)	20,492 (22.9)	19,924 (22.3)	18,832 (21.1)
1–2 times/week	22,442 (25.0)	23,985 (26.8)	24,009 (26.8)	23,404 (26.2)	21,626 (24.2)
1–3 times/month	8,929 (10.0)	10,198 (11.4)	10,379 (11.6)	10,413 (11.6)	9,728 (10.9)
Special occasions only	7,932 (8.8)	9,650 (10.8)	10,612 (11.9)	11,280 (12.6)	11,520 (12.9)
Never	4,905 (5.5)	6,035 (6.7)	6,967 (7.8)	7,867 (8.8)	9,268 (10.4)
Hypertension, yes	47,228 (52.6)	47,418 (52.8)	47,391 (52.8)	45,971 (51.2)	43,542 (48.5)
Antihypertension meds, yes	16,572 (18.5)	17,633 (19.8)	18,015 (20.2)	17,939 (20.2)	17,245 (19.4)
Cholesterol lowering meds, yes	12,826 (14.4)	13,935 (15.6)	14,648 (16.4)	14,894 (16.7)	14,602 (16.5)

Data are mean (SD), Median [IQR], or N (%).

BMI, body mass index; MET, metabolic equivalent task; NO, nitric oxide; NO_2_, nitrogen dioxide; PM, particulate matter; WC, waist circumference.

The associations between individual air pollutants and the risk of T2D are shown in **Table 2**. We found that PM_2.5_, NO_2_, and NO were each associated with an increased risk of T2D in the multivariate-adjusted models, while a positive and marginal association was found for PM_2.5–10_. Compared to individuals exposed to the lowest quintile of air pollutant, the HR for T2D for those exposed to the highest quintile was 1.12 (95% CI: 1.05 to 1.18, *p* = 0.002) for PM_2.5_, 1.11 (1.04 to 1.18, *p* < 0.001) for NO_2_, and 1.12 (1.06 to 1.18, *p* < 0.001) for NO, respectively.

**Table 2:** HRs of T2D by individual air pollutant concentration among 461,191 UK Biobank participants.

	HR (95% CI) per SD increase	Air pollutant concentration in quintiles					*P* for trend
		Q1	Q2	Q3	Q4	Q5	
PM_2.5_	1.04 (1.02, 1.05)	Ref.	1.08 (1.02, 1.14)	1.06 (1.01, 1.12)	1.06 (1.01, 1.12)	1.12 (1.05, 1.18)	0.002
PM_2.5–10_	1.01 (0.99, 1.03)	Ref.	1.02 (0.97, 1.07)	1.04 (0.98, 1.09)	1.01 (0.96, 1.06)	1.06 (1.00, 1.11)	0.081
NO_2_	1.05 (1.02, 1.07)	Ref.	1.04 (0.99, 1.10)	1.07 (1.01, 1.13)	1.11 (1.05, 1.17)	1.11 (1.04, 1.18)	<0.001
NO	1.04 (1.02, 1.05)	Ref.	1.06 (1.00, 1.11)	1.06 (1.01, 1.12)	1.12 (1.06, 1.18)	1.12 (1.06, 1.18)	<0.001

CI, confidence interval; GRS, genetic risk score; HR, hazard ratio; NO, estimated nitric oxide; NO_2_, nitrogen dioxide; PM_2.5_, particulate matter with aerodynamic diameter ≤2.5 μm; PM_2.5–10_, particulate matter with an aerodynamic diameter between 2.5 and 10 μm; T2D, type 2 diabetes.

Models were adjusted for age, ethnicity, sex, Townsend deprivation index, center, alcohol intake, smoking status, physical activity, sedentary hour, healthy diet score, BMI, systolic blood pressure, antihypertension meds, high cholesterol, and T2D GRS.

When jointly considering the 4 air pollutants by the air pollution score, we found that participants exposed to higher levels of the score were significantly associated with a higher risk of T2D (**Table 3**). In the age, ethnicity, and sex-adjusted model (model 1), individuals within the fifth quintile (Q5) of air pollution score had a 71% increased risk of developing T2D, compared to those in Q1 (*p* < 0.001). After further adjustment for assessment center, Townsend deprivation index, alcohol intake, smoking status, total physical activity, sedentary hours, and healthy diet score, the association between air pollution score and risk of T2D attenuated but remained significant (HR for extreme quintiles was 1.16 (1.10 to 1.22, *p* < 0.001) in model 2). Additional adjustment for BMI, systolic blood pressure, antihypertension medication use, high cholesterol, and GRS of T2D did not appreciably change the result (HR for extreme quintiles was 1.12 (1.06 to 1.109, *p* < 0.001) in model 3). Also, the results were stable when we excluded PM_2.5–10_ in the air pollution score (**S4 Table**). Sensitivity analysis excluding T2D cases developed within the first 2 years of follow-up yielded similar results (**S5 Table**). Moreover, the results were robust when only participants living in the current address for at least 5 years were included (**S6 Table**).

**Table 3:** Associations between air pollution score and incident T2D among 461,191 UK Biobank participants.

	Model 1		Model 2		Model 3	
	HR (95% CI)	*p*-value	HR (95% CI)	*p*-value	HR (95% CI)	*p*-value
Air pollution score per SD	1.17 (1.16, 1.19)	<0.001	1.05 (1.03, 1.07)	<0.001	1.04 (1.02, 1.06)	<0.001
Q1	ref	-	ref	-	ref	-
Q2	1.20 (1.14, 1.27)	<0.001	1.07 (1.02, 1.13)	0.007	1.05 (0.99, 1.10)	0.11
Q3	1.34 (1.28, 1.41)	<0.001	1.11 (1.05, 1.16)	<0.001	1.06 (1.00, 1.11)	0.051
Q4	1.51 (1.44, 1.58)	<0.001	1.15 (1.09, 1.21)	<0.001	1.09 (1.03, 1.15)	0.002
Q5	1.71 (1.63, 1.80)	<0.001	1.16 (1.10, 1.22)	<0.001	1.12 (1.06, 1.19)	<0.001

Model 1: adjusted for age, ethnicity, and sex.

Model 2: Model 1+ Townsend deprivation index, center, alcohol intake, smoking status, physical activity, sedentary hour, and healthy diet score.

Model 3: Model 2+ BMI, SBP, antihypertension meds, high cholesterol, and T2D-GRS.

BMI, body mass index; CI, confidence interval; GRS, genetic risk score; HR, hazard ratio; SBP, systolic blood pressure; T2D, type 2 diabetes.

**Fig 2:**
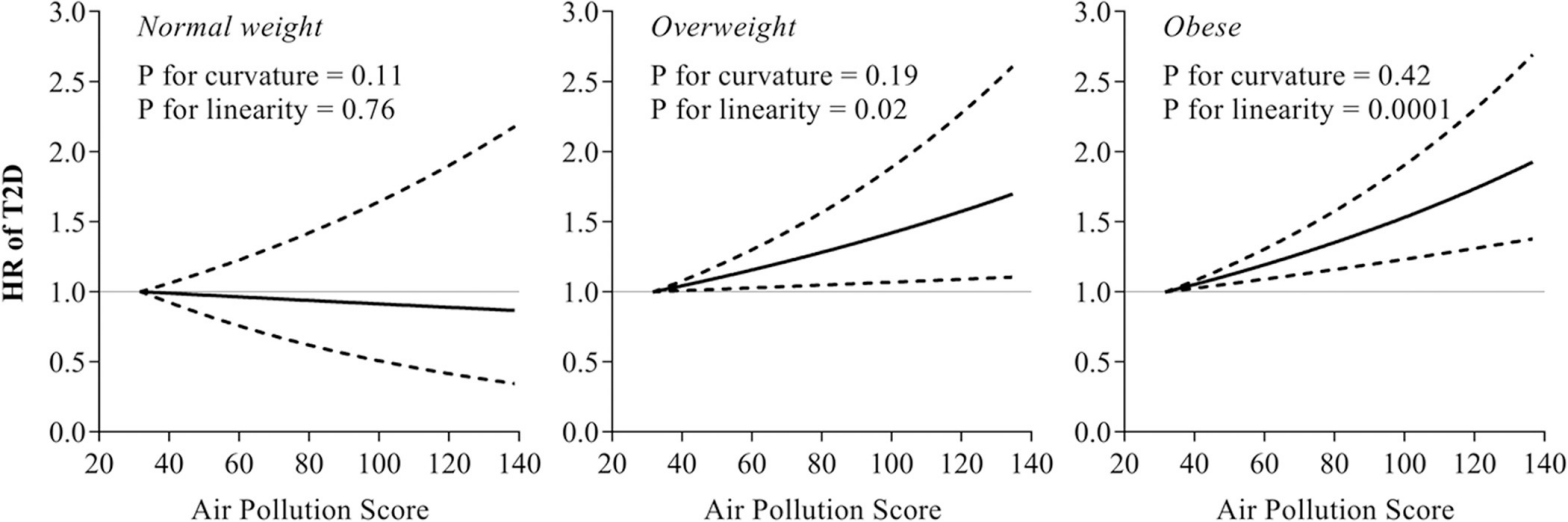
Dose-responsive relationship of air pollution score with T2D incidence according to obesity status. Dashed lines represent the 95% CIs of the HR. Multivariable models were adjusted for age, sex, Townsend deprivation index, center, alcohol intake, smoking status, physical activity, sedentary hours, healthy diet score, systolic blood pressure, antihypertension meds, high cholesterol, and T2D GRS. Sample size for normal weight, overweight, and obese subgroup were 149,945, 193,492, and 105,569, respectively. CI, confidence interval; GRS, genetic risk score; HR, hazard ratio; T2D, type 2 diabetes.

Intriguingly, we found significant interactions between the air pollution score and BMI on the risk of T2D (**S7 Table**; *p*-interaction < 0.001). In the stratified analysis according to the obesity status (normal-weight, overweight, and obesity), the associations between air pollution score and T2D risk appeared to be stronger among overweight and obese participants, while no association was found among those with normal-weight (**Fig 2**). The spline analysis showed a significant linear relationship between air pollution score and the risk of T2D among overweight and obese participants (p for curvature was 0.19 and 0.42, p for linearity was 0.02 and 0.0001, respectively, in the overweight and obese group). A similar interaction pattern was observed between air pollution score and central obesity status (**S1 Fig** and **S7 Table**; *p*-interaction = 0.015). We also further tested the interaction between air pollution score and obesity GRS on the risk of T2D. However, we did not find a significant modification effect by the obesity GRS, though the relationship was statistically significant among participants with a high genetic risk of obesity (**S2 Fig**).

We did not observe a significant interaction between the air pollution score and the genetic risk of T2D, suggesting that such association was consistent regardless of participants’ genetic predisposition to T2D (**S3 Fig**). We noted that the relationship between air pollution score and T2D were stronger among those with intermediate/high genetic predisposition to T2D, though the test for interaction was not significant.

## Discussion

In this large-scale cohort, we found that long-term exposure to various ambient air pollutants including PM_2.5_, PM_2.5–10_, NO_2,_ and NO, individually or jointly as an air pollution score, was significantly associated with an increased risk of T2D in the whole population in a dose-responsive fashion, independent of the traditional risk factors. In addition, we found that the association was significantly modified by obesity status, with more pronounced associations observed among overweight/obese individuals than in normal-weight individuals.

The relationship between air pollution and T2D has only been recently investigated. Our findings on the relations between individual air pollutants and T2D are in line with the results from several cohort studies assessing the long-term exposure to air pollutants [16,20–23,27,40]. A study conducted in Hong Kong (*N* = 57,053) among an elder population (aged 65+) with a mean follow-up of 9.8 years demonstrated that PM_2.5_ was associated with an increased risk of T2D [40]. NO_2_ and NO_x_ were also found to be associated with an increased risk of T2D [16,20,22]. The association between PM_2.5–10_ and T2D was less studied, and the results were inconsistent [14,23,41]. The conflicting findings from the limited body of previous studies are partly accounted for by the varies in study design, population, sample size, air pollutants measurement, as well as exposure duration.

We observed significant associations of the joint exposure to various air pollutants, evaluated by an air pollution score, with the risk of T2D. Evaluating the health impact of multiple air pollutants has been recognized as a priority in the US Environmental Protection Agency’s integrated, cross-disciplinary research planning [17,19]. The recent assessment of public health consequences of air pollution has been encouraged to move toward a multipollutant approach [42]. Indeed, humans are simultaneously exposed to a complex mixture of air pollutants. The air pollution score could reflect a more comprehensive exposure to various air pollutants and acknowledge the importance of assessing the health burden from simultaneous exposure to multiple air pollutants. This simple algorithm is also easy to interpret and facilitate public health protection from air pollution. Our study indicates that various air pollutants, when exposed together, may jointly influence the T2D risk.

Although the underlying mechanisms linking various air pollutants and increased risk of T2D are still unclear, several mechanisms have been proposed. PM is among the most studied air pollutant on the risk of T2D. A main working hypothesis is that PM may induce oxidative stress and subsequent visceral adipose tissue inflammation, which further leads to insulin resistance [43–46]. Other possible mechanisms explaining the association between air pollution and T2D include the disturbed autonomic nervous system [47], epigenetic changes [48,49], mitochondrial dysfunction [50], as well as alterations in the composition and function of the human gut microbiome [51].

Interestingly, we found that being overweight/obese significantly amplified the association between air pollution score and the risk of T2D, while no association was observed among normal-weight participants. Previous evidence in the Danish Nurse Cohort Study also showed that the association between PM_2.5_ and T2D was augmented among obese women [27]. Notably, the increased susceptibility of obesity to the adverse effect of air pollution has also been seen with regard to other diseases, including cardiovascular events [52], chronic obstructive pulmonary disease [53], and hypertension [54], lending support to the modification effects of metabolic status on the relations between air pollution and T2D. Our finding on the interaction between air pollution and obesity status is biologically plausible. A previous study has shown that obesity may enhance associations between air pollution and systemic inflammation [55]. As obesity is a proinflammatory state, being overweight/obese may predispose to a heightened inflammatory response and oxidative stress. When both conditions (air pollution and obesity) are present, there is a multiplicative effect [53,55]. Furthermore, several hormones such as adiponectin, leptin, and resistin, which are inversely related to adiposity, may accentuate the adverse effect of air pollution [25,56,57]. Moreover, changes in respiratory physiology among obese individuals may also explain the enhanced susceptibility to air pollution. Previous particle deposition studies and a weight loss trial have shown an association between obesity and enlarged tidal volume, which lead to a greater dose of inhaled particles among obese participants [58–60].

We did not observe the modification effects by the genetic predisposition to obesity or diabetes on the relationship between air pollution and risk of T2D. The null results were partly due to the small variation of BMI or T2D risk explained by the discovered genetic loci [36]. Further studies with a large sample size and prospective design are warranted to replicate our findings.

To the best of our knowledge, this is the first study to investigate the joint associations of various ambient air pollutants with the risk of T2D. The major strengths of our study include a large sample size, prospective study design of UK Biobank, and comprehensive incorporation of various air pollutants in the air pollution score. However, we acknowledge the current analysis has several potential limitations. First, although we have comprehensively considered various air pollutants, some of the previously reported air pollutants were not available in UK Biobank, such as O_3_, sulfur dioxides, and carbon monoxide. Second, the participants of UK Biobank are mainly of European descent. Whether the observed association could be applied to other ethnic groups and areas warrants further investigation. Third, the observational nature of the study precludes conclusions about causality. Fourth, although we have comprehensively considered typical risk factors and potential confounders, because of the observational study design, residual confounding is inevitable. Lastly, we do not have air pollution data during follow-up.

In conclusion, we found that long-term exposure to various air pollutants, individually or jointly, was associated with an increased risk of T2D, and that the association was more pronounced among overweight/obese participants and central obese participants. Our findings highlight the importance to comprehensively assess the various air pollutants and body weight management in the prevention of T2D.

## Supporting information

S1 STROBE checklist Checklist of items that should be included in reports of *cohort studies*. (DOCX)

S1 Table Information of genetic variants associated with T2D in the UK Biobank study. EA, effect allele; NEA, noneffect allele; OR, odds ratio; SE, standard error; SNP, single nucleotide polymorphism; T2D, type 2 diabetes.
(DOCX)

S2 Table Information of genetic variants associated with BMI in the UK Biobank study. β, beta coefficient; BMI, body mass index; Chr, chromosome; EA, effect allele; NEA, noneffect allele; SE, standard error; SNP, single nucleotide polymorphism.
(DOCX)

S3 Table Pearson correlations between the individual air pollutant incorporated in the air pollution score. *Indicates *p* < 0.001.
(DOCX)

S4 Table Associations between modified air pollution score (by removing PM_2.5–10_) and incident T2D among 461,191 UK Biobank participants. Model 1: adjusted for age, ethnicity, and sex; Model 2: Model 1+ Townsend deprivation index, center, alcohol intake, smoking status, physical activity, sedentary hour, healthy diet score; Model 3: Model 2+ BMI, SBP, antihypertension meds, high cholesterol, and T2D-GRS. BMI, body mass index; GRS, genetic risk score; SBP, systolic blood pressure; T2D, type 2 diabetes.
(DOCX)

S5 Table Associations between air pollution score and incident T2D by excluding T2D cases occurred in the first 2 years of follow-up. Model 1: adjusted for age, ethnicity, and sex; Model 2: Model 1+ Townsend deprivation index, center, alcohol intake, smoking status, physical activity, sedentary hour, healthy diet score; Model 3: Model 2+ BMI, SBP, antihypertension meds, high cholesterol, and T2D-GRS. BMI, body mass index; GRS, genetic risk score; SBP, systolic blood pressure; T2D, type 2 diabetes.
(DOCX)

S6 Table Associations between air pollution score and incident T2D by excluding participants who live in the current address for less than 5 years. Model 1: adjusted for age, ethnicity, and sex; Model 2: Model 1+ Townsend deprivation index, center, alcohol intake, smoking status, physical activity, sedentary hour, healthy diet score; Model 3: Model 2+ BMI, SBP, antihypertension meds, high cholesterol, and T2D-GRS. BMI, body mass index; GRS, genetic risk score; SBP, systolic blood pressure; T2D, type 2 diabetes.
(DOCX)

S7 Table Association between the air pollution score and T2D risk according to general obesity and central obesity. Multivariable models were adjusted for age, sex, Townsend deprivation index, center, alcohol intake, smoking status, physical activity, sedentary hours, healthy diet score, systolic blood pressure, antihypertension meds, high cholesterol, and T2D GRS. GRS, genetic risk score; T2D, type 2 diabetes.
(DOCX)

S1 Fig Dose–response relationship of air pollution score with T2D incidence according to central obesity status. Dashed lines represent the 95% CIs of the HR. Multivariable models were adjusted for age, sex, Townsend deprivation index, center, alcohol intake, smoking status, physical activity, sedentary hours, healthy diet score, systolic blood pressure, antihypertension meds, high cholesterol, and T2D GRS. Sample size for noncentral obese and central obese subgroup were 177,791 and 271,215, respectively. CI, confidence interval; GRS, genetic risk score; HR, hazard ratio; T2D, type 2 diabetes.
(TIF)

S2 Fig Adjusted HR for T2D according to obesity GRS. Stratified analyses were performed by tertiles of obesity GRS. Multivariable models were adjusted for age, sex, Townsend deprivation index, center, alcohol intake, smoking status, physical activity, sedentary hours, healthy diet score, BMI, systolic blood pressure, antihypertension meds, and high cholesterol. BMI, body mass index; CI, confidence interval; GRS, genetic risk score; HR, hazard ratio; T2D, type 2 diabetes.
(TIF)

S3 Fig Adjusted HR for T2D according to T2D GRS. Stratified analyses were performed by tertiles of T2D GRS. Multivariable models were adjusted for age, sex, Townsend deprivation index, center, alcohol intake, smoking status, physical activity, sedentary hours, healthy diet score, BMI, systolic blood pressure, antihypertension meds, and high cholesterol. BMI, body mass index; CI, confidence interval; GRS, genetic risk score; HR, hazard ratio; T2D, type 2 diabetes.
(TIF)

## Abbreviations

BMI: body mass index
CI: confidence interval
ESCAPE: European Study of Cohorts for Air Pollution Effects
GIS: Geographic Information System
GRS: genetic risk score
HR: hazard ratio
IDF: International Diabetes Federation
IPAQ: International Physical Activity Questionnaire
LUR: Land Use Regression
MET: metabolic equivalent task
NO: nitric oxide
NO_2_: nitrogen dioxide
PM: particulate matter
SNP: single nucleotide polymorphism
T2D: type 2 diabetes
WC: waist circumference
