# Supplementary material for: Obesity and the relation between joint exposure to ambient air pollutants and incident type 2 diabetes: A cohort study in UK Biobank
Source: PLoS Med. 2021 Aug 30;18(8):e1003767. doi: 10.1371/journal.pmed.1003767 (PMC8439461; doi:10.1371/journal.pmed.1003767)
Supplement: S1 Table — EA, effect allele; NEA, noneffect allele; OR, odds ratio; SE, standard error; SNP, single nucleotide polymorphism; T2D, type 2 diabetes. (DOCX) [file pmed.1003767.s002.docx]

S1 Table. Information of genetic variants associated with type 2 diabetes in the UK Biobank study

| SNP | Chromosome | Nearest Gene | EA/NEA | OR (SE) |
| --- | --- | --- | --- | --- |
| rs3768321 | 1 | *MACF1* | T/G | 1.144 (0.028) |
| rs12031920 | 1 | *FAF1* | T/A | 1.004 (0.024) |
| rs67156297 | 1 | *ATP8B2* | A/G | 1.041 (0.026) |
| rs340874 | 1 | *PROX1* | C/T | 1.092 (0.024) |
| rs145819220 | 2 | *GCKR* | C/G | 1.122 (0.173) |
| rs6757251 | 2 | *THADA* | C/T | 1.172 (0.04) |
| rs9309245 | 2 | *ASB3* | C/G | 1.019 (0.025) |
| rs1116357 | 2 | *CCDC85A* | G/A | 1.026 (0.023) |
| rs10193447 | 2 | *BCL11A* | T/C | 1.015 (0.024) |
| rs6723108 | 2 | *TMEM163* | T/G | 1.008 (0.023) |
| rs7560163 | 2 | *RBM43/RND3* | C/G | 1.082 (0.24) |
| rs1563575 | 2 | *RBMS1* | G/A | 1.006 (0.026) |
| rs28584669 | 2 | *GRB14* | T/C | 1.031 (0.032) |
| rs1861612 | 2 | *DNER* | A/G | 1.025 (0.023) |
| rs11712037 | 3 | *PPARG* | C/G | 1.108 (0.037) |
| rs35352848 | 3 | *UBE2E2* | T/C | 1.14 (0.03) |
| rs79819696 | 3 | *PSMD6* | A/G | 1.019 (0.236) |
| rs7428936 | 3 | *ADAMTS9* | T/C | 1.064 (0.024) |
| rs11708067 | 3 | *ADCY5* | A/G | 1.096 (0.028) |
| rs4402960 | 3 | *IGF2BP2* | T/G | 1.225 (0.024) |
| rs9820223 | 3 | *ST6GAL1* | C/T | 1.07 (0.024) |
| rs6777684 | 3 | *LPP* | G/A | 1.116 (0.024) |
| rs1531583 | 4 | *MAEA* | T/G | 1.163 (0.057) |
| rs3821943 | 4 | *WFS1* | T/C | 1.067 (0.024) |
| rs7660590 | 4 | *TMEM154* | C/T | 1.015 (0.026) |
| rs60780116 | 4 | *ACSL1* | T/C | 1.046 (0.033) |
| rs173964 | 5 | *ANKRD55* | G/A | 1.077 (0.027) |
| rs9687833 | 5 | *ANKRD55* | A/G | 1.088 (0.028) |
| rs6453287 | 5 | *ZBED3* | C/A | 1.06 (0.025) |
| rs78408340 | 5 | *PAM* | G/C | 1.637 (0.097) |
| rs74944275 | 5 | *PAM* | T/C | 1.092 (0.053) |
| rs6923241 | 6 | *SSR1/RREB1* | T/C | 1.003 (0.026) |
| rs7451008 | 6 | *CDKAL1* | C/T | 1.154 (0.026) |
| rs2244020 | 6 | *HLA-B* | G/A | 1.047 (0.025) |
| rs143308245 | 6 | *ZFAND3* | T/A | 1.744 (0.613) |
| rs11759026 | 6 | *CENPW* | G/A | 1.108 (0.027) |
| rs10276674 | 7 | *DGKB* | C/T | 1.114 (0.029) |
| rs10238625 | 7 | *DGKB* | A/G | 1.074 (0.024) |
| rs1635852 | 7 | *JAZF1* | T/C | 1.087 (0.023) |
| rs878521 | 7 | *GCK* | A/G | 1.075 (0.027) |
| rs10229583 | 7 | *PAX4* | A/G | 1.007 (0.027) |
| rs73455744 | 7 | *GCC1* | G/A | 1.268 (1.024) |
| rs791595 | 7 | *MIR129-LEP* | G/A | 1.051 (0.031) |
| rs10954284 | 7 | *KLF14* | T/A | 1.089 (0.023) |
| rs1182436 | 7 | *MNX1* | C/T | 1.129 (0.031) |
| rs516946 | 8 | *ANK1* | C/T | 1.085 (0.028) |
| rs4734285 | 8 | *TP53INP1* | T/C | 1.038 (0.024) |
| rs11786613 | 8 | *TP53INP1* | C/A | 1.195 (0.089) |
| rs3802177 | 8 | *SLC30A8* | G/A | 1.146 (0.026) |
| rs10758593 | 9 | *GLIS3* | A/G | 1.086 (0.024) |
| rs10965223 | 9 | *CDKN2A/B* | A/G | 1.049 (0.024) |
| rs10965248 | 9 | *CDKN2A/B* | T/C | 1.21 (0.032) |
| rs10757282 | 9 | *CDKN2A/B* | C/T | 1.014 (0.024) |
| rs1575972 | 9 | *DMRTA1* | T/A | 1.042 (0.067) |
| rs13301067 | 9 | *TLE4* | G/A | 1.175 (0.051) |
| rs9410573 | 9 | *TLE1* | T/C | 1.123 (0.024) |
| rs635634 | 9 | *ABO* | T/C | 1.013 (0.03) |
| rs11787792 | 9 | *GPSM1* | A/G | 1.052 (0.025) |
| rs10998572 | 10 | *VPS26A* | C/A | 1.056 (0.049) |
| rs810517 | 10 | *ZMIZ1* | C/T | 1.118 (0.023) |
| rs11187140 | 10 | *HHEX/IDE* | G/A | 1.172 (0.025) |
| rs7903146 | 10 | *TCF7L2* | T/C | 1.501 (0.024) |
| rs10886471 | 10 | *GRK5* | T/C | 1.014 (0.023) |
| rs2292626 | 10 | *PLEKHA1* | C/T | 1.078 (0.023) |
| rs2334499 | 11 | *DUSP8* | T/C | 1.041 (0.024) |
| rs11564732 | 11 | *INS-IGF2* | C/T | 1.068 (0.089) |
| rs7107784 | 11 | *MIR4686* | G/A | 1.062 (0.025) |
| rs231360 | 11 | *KCNQ1* | T/C | 1.084 (0.024) |
| rs233449 | 11 | *KCNQ1* | G/A | 1.114 (0.027) |
| rs2237897 | 11 | *KCNQ1* | C/T | 1.35 (0.066) |
| rs441613 | 11 | *KCNQ1* | C/T | 1.009 (0.025) |
| rs5219 | 11 | *KCNJ11* | T/C | 1.072 (0.024) |
| rs1061810 | 11 | *HSD17B12* | A/C | 1.025 (0.025) |
| rs76550717 | 11 | *ARAP1 (CENTD2)* | A/G | 1.12 (0.033) |
| rs10830963 | 11 | *MTNR1B* | G/C | 1.119 (0.026) |
| rs11063018 | 12 | *CCND2* | C/T | 1.057 (0.03) |
| rs188827514 | 12 | *CCND2* | A/G | 1.481 (0.183) |
| rs4238013 | 12 | *CCND2* | C/T | 1.108 (0.028) |
| rs7953190 | 12 | *KLHDC5* | T/C | 1.125 (0.03) |
| rs147538848 | 12 | *FAM60A* | G/A | 1.24 (1.024) |
| rs2258238 | 12 | *HMGA2* | T/A | 1.104 (0.037) |
| rs6581998 | 12 | *TSPAN8/LGR5* | C/T | 1.028 (0.026) |
| rs56348580 | 12 | *HNF1A (TCF1)* | G/C | 1.048 (0.026) |
| rs2851437 | 12 | *MPHOSPH9* | A/C | 1.025 (0.028) |
| rs9552911 | 13 | *SGCG* | G/A | 1.214 (0.464) |
| rs7330796 | 13 | *TBC1D4* | T/C | 1.023 (0.036) |
| rs11616380 | 13 | *SPRY2* | G/T | 1.153 (0.027) |
| rs10146997 | 14 | *NRXN3* | G/A | 1.005 (0.028) |
| rs67839313 | 15 | *INAFM2* | C/T | 1.032 (0.037) |
| rs4774420 | 15 | *C2CD4A* | C/T | 1.008 (0.026) |
| rs952471 | 15 | *HMG20A* | G/C | 1.065 (0.026) |
| rs62006309 | 15 | *ZFAND6* | A/G | 1.03 (0.023) |
| rs12595616 | 15 | *PRC1* | C/T | 1.066 (0.024) |
| rs1558902 | 16 | *FTO* | A/T | 1.059 (0.023) |
| rs8056814 | 16 | *BCAR1* | G/A | 1.225 (0.045) |
| rs2925979 | 16 | *CMIP* | T/C | 1.059 (0.025) |
| rs9911305 | 17 | *SRR* | A/G | 1.07 (0.026) |
| rs7224685 | 17 | *ZZEF1* | T/G | 1.037 (0.025) |
| rs13342692 | 17 | *SLC16A11/A13* | T/C | 1.037 (0.127) |
| rs78761021 | 17 | *GLP2R* | G/A | 1.039 (0.025) |
| rs757209 | 17 | *HNF1B (TCF2)* | G/A | 1.061 (0.024) |
| rs7234111 | 18 | *LAMA1* | C/T | 1.083 (0.024) |
| rs1942880 | 18 | *MC4R* | T/C | 1.014 (0.025) |
| rs79851087 | 18 | *MC4R* | A/G | 1.004 (0.087) |
| rs12454712 | 18 | *BCL2A* | T/C | 1.069 (0.024) |
| rs58489806 | 19 | *CILP2* | T/C | 1.125 (0.04) |
| rs139990642 | 19 | *PEPD* | A/G | 1.229 (0.145) |
| rs429358 | 19 | *APOE* | T/C | 1.067 (0.033) |
| rs55864746 | 19 | *GIPR* | A/G | 1.11 (0.026) |
| rs12625671 | 20 | *HNF4A* | C/T | 1.081 (0.037) |
| rs1800961 | 20 | *HNF4A* | T/C | 1.322 (0.06) |
| rs2023681 | 22 | *MTMR3/HORMAD2* | G/A | 1.132 (0.042) |

SNP, single nucleotide polymorphism; EA, effect allele; NEA, non-effect allele; OR, odds ratio; SE, standard error.
