# Supplementary material for: Obesity and the relation between joint exposure to ambient air pollutants and incident type 2 diabetes: A cohort study in UK Biobank
Source: PLoS Med. 2021 Aug 30;18(8):e1003767. doi: 10.1371/journal.pmed.1003767 (PMC8439461; doi:10.1371/journal.pmed.1003767)
Supplement: S2 Table — β, beta coefficient; BMI, body mass index; Chr, chromosome; EA, effect allele; NEA, noneffect allele; SE, standard error; SNP, single nucleotide polymorphism. (DOCX) [file pmed.1003767.s003.docx]

S2 Table. Information of genetic variants associated with BMI in the UK Biobank study

| SNP | Chr | Nearest Gene | EA/NEA | β (SE) | P |
| --- | --- | --- | --- | --- | --- |
| rs1000940 | 17 | *RABEP1* | G/A | 0.0192 (0.0034) | 1.28E-08 |
| rs10132280 | 14 | *STXBP6* | C/A | 0.023 (0.0034) | 1.14E-11 |
| rs1016287 | 2 | *FLJ30838* | T/C | 0.0229 (0.0034) | 2.25E-11 |
| rs10182181 | 2 | *ADCY3* | G/A | 0.0307 (0.0031) | 8.78E-24 |
| rs10733682 | 9 | *LMX1B* | A/G | 0.0174 (0.0031) | 1.83E-08 |
| rs10938397 | 4 | *GNPDA2* | G/A | 0.0402 (0.0031) | 3.21E-38 |
| rs10968576 | 9 | *LINGO2* | G/A | 0.0249 (0.0033) | 6.61E-14 |
| rs11030104 | 11 | *BDNF* | A/G | 0.0414 (0.0038) | 5.56E-28 |
| rs11057405 | 12 | *CLIP1* | G/A | 0.0307 (0.0055) | 2.02E-08 |
| rs11126666 | 2 | *KCNK3* | A/G | 0.0207 (0.0034) | 1.33E-09 |
| rs11165643 | 1 | *PTBP2* | T/C | 0.0218 (0.0031) | 2.07E-12 |
| rs11191560 | 10 | *NT5C2* | C/T | 0.0308 (0.0053) | 8.45E-09 |
| rs11583200 | 1 | *ELAVL4* | C/T | 0.0177 (0.0031) | 1.48E-08 |
| rs1167827 | 7 | *HIP1* | G/A | 0.0202 (0.0033) | 6.33E-10 |
| rs11688816 | 2 | *EHBP1* | G/A | 0.0172 (0.0031) | 1.89E-08 |
| rs11727676 | 4 | *HHIP* | T/C | 0.0358 (0.0064) | 2.55E-08 |
| rs11847697 | 14 | *PRKD1* | T/C | 0.0492 (0.0084) | 3.99E-09 |
| rs12016871 | 13 | *MTIF3* | T/C | 0.0298 (0.0047) | 2.29E-10 |
| rs12286929 | 11 | *CADM1* | G/A | 0.0217 (0.0031) | 1.31E-12 |
| rs12401738 | 1 | *FUBP1* | A/G | 0.0211 (0.0033) | 1.15E-10 |
| rs12429545 | 13 | *OLFM4* | A/G | 0.0334 (0.0047) | 1.09E-12 |
| rs12446632 | 16 | *GPRC5B* | G/A | 0.0403 (0.0046) | 1.48E-18 |
| rs12566985 | 1 | *FPGT-TNNI3K* | G/A | 0.0242 (0.0031) | 3.28E-15 |
| rs12885454 | 14 | *PRKD1* | C/A | 0.0207 (0.0033) | 1.94E-10 |
| rs12940622 | 17 | *RPTOR* | G/A | 0.0182 (0.0031) | 2.49E-09 |
| rs13021737 | 2 | *TMEM18* | G/A | 0.0601 (0.004) | 1.11E-50 |
| rs13078960 | 3 | *CADM2* | G/T | 0.0297 (0.0039) | 1.74E-14 |
| rs13107325 | 4 | *SLC39A8* | T/C | 0.0477 (0.0068) | 1.83E-12 |
| rs13191362 | 6 | *PARK2* | A/G | 0.0277 (0.0048) | 7.34E-09 |
| rs13201877 | 6 | *IFNGR1* | G/A | 0.0233 (0.0045) | 2.35E-07 |
| rs1441264 | 13 | *MIR548A2* | A/G | 0.0175 (0.0032) | 6.04E-08 |
| rs1460676 | 2 | *FIGN* | C/T | 0.0197 (0.004) | 8.98E-07 |
| rs1516725 | 3 | *ETV5* | C/T | 0.0451 (0.0046) | 1.89E-22 |
| rs1528435 | 2 | *UBE2E3* | T/C | 0.0178 (0.0031) | 1.2E-08 |
| rs1558902 | 16 | *FTO* | A/T | 0.0818 (0.0031) | 7.5E-153 |
| rs16851483 | 3 | *RASA2* | T/G | 0.0483 (0.0077) | 3.55E-10 |
| rs16907751 | 8 | *ZBTB10* | C/T | 0.035 (0.0066) | 1.26E-07 |
| rs16951275 | 15 | *MAP2K5* | T/C | 0.0311 (0.0037) | 1.91E-17 |
| rs17001654 | 4 | *SCARB2* | G/C | 0.0306 (0.0053) | 7.76E-09 |
| rs17024393 | 1 | *GNAT2* | C/T | 0.0658 (0.0088) | 7.03E-14 |
| rs17094222 | 10 | *HIF1AN* | C/T | 0.0249 (0.0038) | 5.94E-11 |
| rs17203016 | 2 | *CREB1* | G/A | 0.021 (0.0039) | 8.15E-08 |
| rs17405819 | 8 | *HNF4G* | T/C | 0.0224 (0.0033) | 2.07E-11 |
| rs17724992 | 19 | *PGPEP1* | A/G | 0.0194 (0.0035) | 3.42E-08 |
| rs1808579 | 18 | *C18orf8* | C/T | 0.0167 (0.0031) | 4.17E-08 |
| rs1928295 | 9 | *TLR4* | T/C | 0.0188 (0.0031) | 7.91E-10 |
| rs2033529 | 6 | *TDRG1* | G/A | 0.019 (0.0033) | 1.39E-08 |
| rs2033732 | 8 | *RALYL* | C/T | 0.0192 (0.0035) | 4.89E-08 |
| rs205262 | 6 | *C6orf106* | G/A | 0.0221 (0.0035) | 1.75E-10 |
| rs2075650 | 19 | *TOMM40* | A/G | 0.0258 (0.0045) | 1.25E-08 |
| rs2080454 | 16 | *CBLN1* | C/A | 0.0168 (0.0031) | 6.55E-08 |
| rs2112347 | 5 | *POC5* | T/G | 0.0261 (0.0031) | 6.19E-17 |
| rs2121279 | 2 | *LRP1B* | T/C | 0.0245 (0.0044) | 2.31E-08 |
| rs2176040 | 2 | *LOC646736* | A/G | 0.0141 (0.0031) | 6.06E-06 |
| rs2176598 | 11 | *HSD17B12* | T/C | 0.0198 (0.0036) | 2.97E-08 |
| rs2207139 | 6 | *TFAP2B* | G/A | 0.0447 (0.004) | 4.13E-29 |
| rs2245368 | 7 | *PMS2L11* | C/T | 0.0317 (0.0057) | 3.19E-08 |
| rs2287019 | 19 | *QPCTL* | C/T | 0.036 (0.0042) | 4.59E-18 |
| rs2365389 | 3 | *FHIT* | C/T | 0.02 (0.0031) | 1.63E-10 |
| rs2650492 | 16 | *SBK1* | A/G | 0.0207 (0.0035) | 1.92E-09 |
| rs2820292 | 1 | *NAV1* | C/A | 0.0195 (0.0031) | 1.83E-10 |
| rs2836754 | 21 | *ETS2* | C/T | 0.0164 (0.0032) | 4.16E-07 |
| rs29941 | 19 | *KCTD15* | G/A | 0.0182 (0.0033) | 2.41E-08 |
| rs3101336 | 1 | *NEGR1* | C/T | 0.0334 (0.0031) | 2.66E-26 |
| rs3736485 | 15 | *DMXL2* | A/G | 0.0176 (0.0031) | 7.41E-09 |
| rs3810291 | 19 | *ZC3H4* | A/G | 0.0283 (0.0036) | 4.81E-15 |
| rs3817334 | 11 | *MTCH2* | T/C | 0.0262 (0.0031) | 5.15E-17 |
| rs3849570 | 3 | *GBE1* | A/C | 0.0188 (0.0034) | 2.6E-08 |
| rs3888190 | 16 | *ATP2A1* | A/C | 0.0309 (0.0031) | 3.14E-23 |
| rs4256980 | 11 | *TRIM66* | G/C | 0.0209 (0.0031) | 2.9E-11 |
| rs4740619 | 9 | *C9orf93* | T/C | 0.0179 (0.0031) | 4.56E-09 |
| rs4787491 | 16 | *INO80E* | G/A | 0.0159 (0.0034) | 2.24E-06 |
| rs492400 | 2 | *USP37* | C/T | 0.0158 (0.0031) | 4.17E-07 |
| rs543874 | 1 | *SEC16B* | G/A | 0.0482 (0.0039) | 2.62E-35 |
| rs6091540 | 20 | *ZFP64* | C/T | 0.0188 (0.0035) | 8.02E-08 |
| rs6465468 | 7 | *ASB4* | T/G | 0.0166 (0.0035) | 2.32E-06 |
| rs6477694 | 9 | *EPB41L4B* | C/T | 0.0174 (0.0031) | 2.67E-08 |
| rs6567160 | 18 | *MC4R* | C/T | 0.0556 (0.0036) | 3.93E-53 |
| rs657452 | 1 | *AGBL4* | A/G | 0.0227 (0.0031) | 5.48E-13 |
| rs6804842 | 3 | *RARB* | G/A | 0.0185 (0.0031) | 2.48E-09 |
| rs7138803 | 12 | *BCDIN3D* | A/G | 0.0315 (0.0031) | 8.15E-24 |
| rs7141420 | 14 | *NRXN3* | T/C | 0.0235 (0.0031) | 1.23E-14 |
| rs7164727 | 15 | *LOC100287559* | T/C | 0.018 (0.0033) | 6.83E-08 |
| rs7239883 | 18 | *LOC284260* | G/A | 0.0164 (0.0031) | 1.63E-07 |
| rs7243357 | 18 | *GRP* | T/G | 0.0217 (0.004) | 3.86E-08 |
| rs758747 | 16 | *NLRC3* | T/C | 0.0225 (0.0037) | 7.47E-10 |
| rs7599312 | 2 | *ERBB4* | G/A | 0.022 (0.0034) | 1.17E-10 |
| rs7715256 | 5 | *GALNT10* | G/T | 0.0163 (0.0031) | 1.7E-07 |
| rs7899106 | 10 | *GRID1* | G/A | 0.0395 (0.0071) | 2.96E-08 |
| rs7903146 | 10 | *TCF7L2* | C/T | 0.0234 (0.0034) | 1.11E-11 |
| rs9374842 | 6 | *LOC285762* | T/C | 0.0187 (0.0035) | 9.67E-08 |
| rs9400239 | 6 | *FOXO3* | C/T | 0.0188 (0.0033) | 1.61E-08 |
| rs9540493 | 13 | *MIR548X2* | A/G | 0.0172 (0.0033) | 1.42E-07 |
| rs9641123 | 7 | *CALCR* | C/G | 0.0191 (0.0038) | 5E-07 |
| rs977747 | 1 | *TAL1* | T/G | 0.0167 (0.0031) | 8.65E-08 |
| rs9914578 | 17 | *SMG6* | G/C | 0.0201 (0.0038) | 8.99E-08 |
| rs9925964 | 16 | *KAT8* | A/G | 0.0192 (0.0031) | 8.11E-10 |

SNP, single nucleotide polymorphism; Chr, chromosome; EA, effect allele; NEA, non-effect allele; β, beta coefficient; SE, standard error
