# Supplementary material for: Obesity and the relation between joint exposure to ambient air pollutants and incident type 2 diabetes: A cohort study in UK Biobank
Source: PLoS Med. 2021 Aug 30;18(8):e1003767. doi: 10.1371/journal.pmed.1003767 (PMC8439461; doi:10.1371/journal.pmed.1003767)
Supplement: S3 Table — *Indicates p < 0.001. (DOCX) [file pmed.1003767.s004.docx]

S3 Table. Pearson correlations between the individual air pollutant incorporated in the air pollution score.

| Air pollutant | PM_2.5_ | PM_2.5-10_ | NO_2_ | NO |
| --- | --- | --- | --- | --- |
| PM_2.5_ | 1.00 | - | - | - |
| PM_2.5-10_ | 0.22* | 1.00 | - | - |
| NO_2_ | 0.86* | 0.20* | 1.00 | - |
| NO | 0.73* | 0.24* | 0.75* | 1.00 |

*Indicates p<0.001.
