# Supplementary material for: Obesity and the relation between joint exposure to ambient air pollutants and incident type 2 diabetes: A cohort study in UK Biobank
Source: PLoS Med. 2021 Aug 30;18(8):e1003767. doi: 10.1371/journal.pmed.1003767 (PMC8439461; doi:10.1371/journal.pmed.1003767)
Supplement: S4 Table — Model 1: adjusted for age, ethnicity, and sex; Model 2: Model 1+ Townsend deprivation index, center, alcohol intake, smoking status, physical activity, sedentary hour, healthy diet score; Model 3: Model 2+ BMI, SBP, antihypertension meds, high cholesterol, and T2D-GRS. BMI, body mass index; GRS, genetic risk score; SBP, systolic blood pressure; T2D, type 2 diabetes. (DOCX) [file pmed.1003767.s005.docx]

S4 Table. Associations between modified air pollution score (by removing PM_2.5-10_) and incident T2D among 461,191 UK Biobank participants.

|  | Model 1 | |  | Model 2 | |  | Model 3 | |
| --- | --- | --- | --- | --- | --- | --- | --- | --- |
|  | HR (95% CI) | p-value |  | HR (95% CI) | p-value |  | HR (95% CI) | p-value |
| Air pollution score per SD | 1.18 (1.16, 1.19) | <0.001 |  | 1.05 (1.03, 1.07) | <0.001 |  | 1.04 (1.02, 1.06) | <0.001 |
| Q1 | Ref. | - |  | Ref. | - |  | Ref. | - |
| Q2 | 1.19 (1.13, 1.25) | <0.001 |  | 1.07 (1.01, 1.12) | 0.015 |  | 1.04 (0.99, 1.10) | 0.14 |
| Q3 | 1.35 (1.29, 1.42) | <0.001 |  | 1.11 (1.06, 1.17) | <0.001 |  | 1.06 (1.01, 1.12) | 0.024 |
| Q4 | 1.50 (1.43, 1.57) | <0.001 |  | 1.14 (1.08, 1.20) | <0.001 |  | 1.08 (1.03, 1.15) | 0.004 |
| Q5 | 1.69 (1.61, 1.78) | <0.001 |  | 1.14 (1.08, 1.20) | <0.001 |  | 1.11 (1.05, 1.18) | <0.001 |

Model 1: adjusted for age, ethnicity, and sex;

Model 2: Model 1+ Townsend deprivation index, center, alcohol intake, smoking status, physical activity, sedentary hour, healthy diet score;

Model 3: Model 2+ BMI, SBP, anti-hypertension meds, high cholesterol, and T2D-GRS.
