# Supplementary material for: Obesity and the relation between joint exposure to ambient air pollutants and incident type 2 diabetes: A cohort study in UK Biobank
Source: PLoS Med. 2021 Aug 30;18(8):e1003767. doi: 10.1371/journal.pmed.1003767 (PMC8439461; doi:10.1371/journal.pmed.1003767)
Supplement: S5 Table — Model 1: adjusted for age, ethnicity, and sex; Model 2: Model 1+ Townsend deprivation index, center, alcohol intake, smoking status, physical activity, sedentary hour, healthy diet score; Model 3: Model 2+ BMI, SBP, antihypertension meds, high cholesterol, and T2D-GRS. BMI, body mass index; GRS, genetic risk score; SBP, systolic blood pressure; T2D, type 2 diabetes. (DOCX) [file pmed.1003767.s006.docx]

S5 Table. Associations between air pollution score and incident T2D by excluding T2D cases occurred in the first 2 years of follow-up.

|  | Model 1 | |  | Model 2 | |  | Model 3 | |
| --- | --- | --- | --- | --- | --- | --- | --- | --- |
|  | HR (95% CI) | p-value |  | HR (95% CI) | p-value |  | HR (95% CI) | p-value |
| Air pollution score, per SD | 1.17 (1.16, 1.19) | <0.001 |  | 1.06 (1.04, 1.08) | <0.001 |  | 1.05 (1.03, 1.07) | <0.001 |
| Q1 | Ref. |  |  | Ref. |  |  | Ref. |  |
| Q2 | 1.19 (1.12, 1.25) | <0.001 |  | 1.07 (1.01, 1.13) | 0.028 |  | 1.04 (0.98, 1.10) | 0.26 |
| Q3 | 1.31 (1.24, 1.39) | <0.001 |  | 1.10 (1.04, 1.17) | 0.001 |  | 1.05 (0.99, 1.11) | 0.14 |
| Q4 | 1.47 (1.39, 1.55) | <0.001 |  | 1.15 (1.08, 1.21) | <0.001 |  | 1.09 (1.02, 1.16) | 0.007 |
| Q5 | 1.71 (1.62, 1.80) | <0.001 |  | 1.19 (1.12, 1.26) | <0.001 |  | 1.14 (1.07, 1.22) | <0.001 |

Model 1: adjusted for age, ethnicity, and sex;

Model 2: Model 1+ Townsend deprivation index, center, alcohol intake, smoking status, physical activity, sedentary hour, healthy diet score;

Model 3: Model 2+ BMI, SBP, anti-hypertension meds, high cholesterol, and T2D-GRS.
