# Supplementary material for: Obesity and the relation between joint exposure to ambient air pollutants and incident type 2 diabetes: A cohort study in UK Biobank
Source: PLoS Med. 2021 Aug 30;18(8):e1003767. doi: 10.1371/journal.pmed.1003767 (PMC8439461; doi:10.1371/journal.pmed.1003767)
Supplement: S7 Table — Multivariable models were adjusted for age, sex, Townsend deprivation index, center, alcohol intake, smoking status, physical activity, sedentary hours, healthy diet score, systolic blood pressure, antihypertension meds, high cholesterol, and T2D GRS. GRS, genetic risk score; T2D, type 2 diabetes. (DOCX) [file pmed.1003767.s008.docx]

S7 Table. Association between the air pollution score and T2D risk according to general obesity and central obesity.

|  | Air pollution score | | | | | per SD | p-int |
| --- | --- | --- | --- | --- | --- | --- | --- |
|  | Q1 | Q2 | Q3 | Q4 | Q5 |  |  |
| Obesity status |  |  |  |  |  |  |  |
| Normal weight | ref | 1.30 (1.09, 1.55) | 1.06 (0.88, 1.28) | 1.18 (0.98, 1.42) | 1.10 (0.90, 1.35) | 0.99 (0.93, 1.05) | <0.001 |
| Overweight | ref | 1.00 (0.91, 1.09) | 1.05 (0.96, 1.15) | 1.09 (0.99, 1.19) | 1.10 (0.99, 1.21) | 1.04 (1.01, 1.08) |  |
| Obese | ref | 1.03 (0.96, 1.10) | 1.04 (0.97, 1.11) | 1.08 (1.01, 1.16) | 1.15 (1.06, 1.24) | 1.05 (1.03, 1.08) |  |
| Central obesity |  |  |  |  |  |  |  |
| No | ref | 1.02 (0.88, 1.18) | 1.03 (0.88, 1.20) | 1.13 (0.97, 1.32) | 1.02 (0.86, 1.21) | 1.00 (0.94, 1.05) | 0.015 |
| Yes | ref | 1.05 (0.99, 1.11) | 1.05 (0.99, 1.11) | 1.09 (1.03, 1.16) | 1.13 (1.06, 1.20) | 1.05 (1.03, 1.07) |  |

Multivariable models were adjusted for age, sex, Townsend deprivation index, center, alcohol intake, smoking status, physical activity, sedentary hours, healthy diet score, systolic blood pressure, anti-hypertension meds, high cholesterol, and T2D genetic risk score
