# Supplementary figures and images for: Obesity and the relation between joint exposure to ambient air pollutants and incident type 2 diabetes: A cohort study in UK Biobank
Source: PLoS Med. 2021 Aug 30;18(8):e1003767. doi: 10.1371/journal.pmed.1003767 (PMC8439461; doi:10.1371/journal.pmed.1003767)

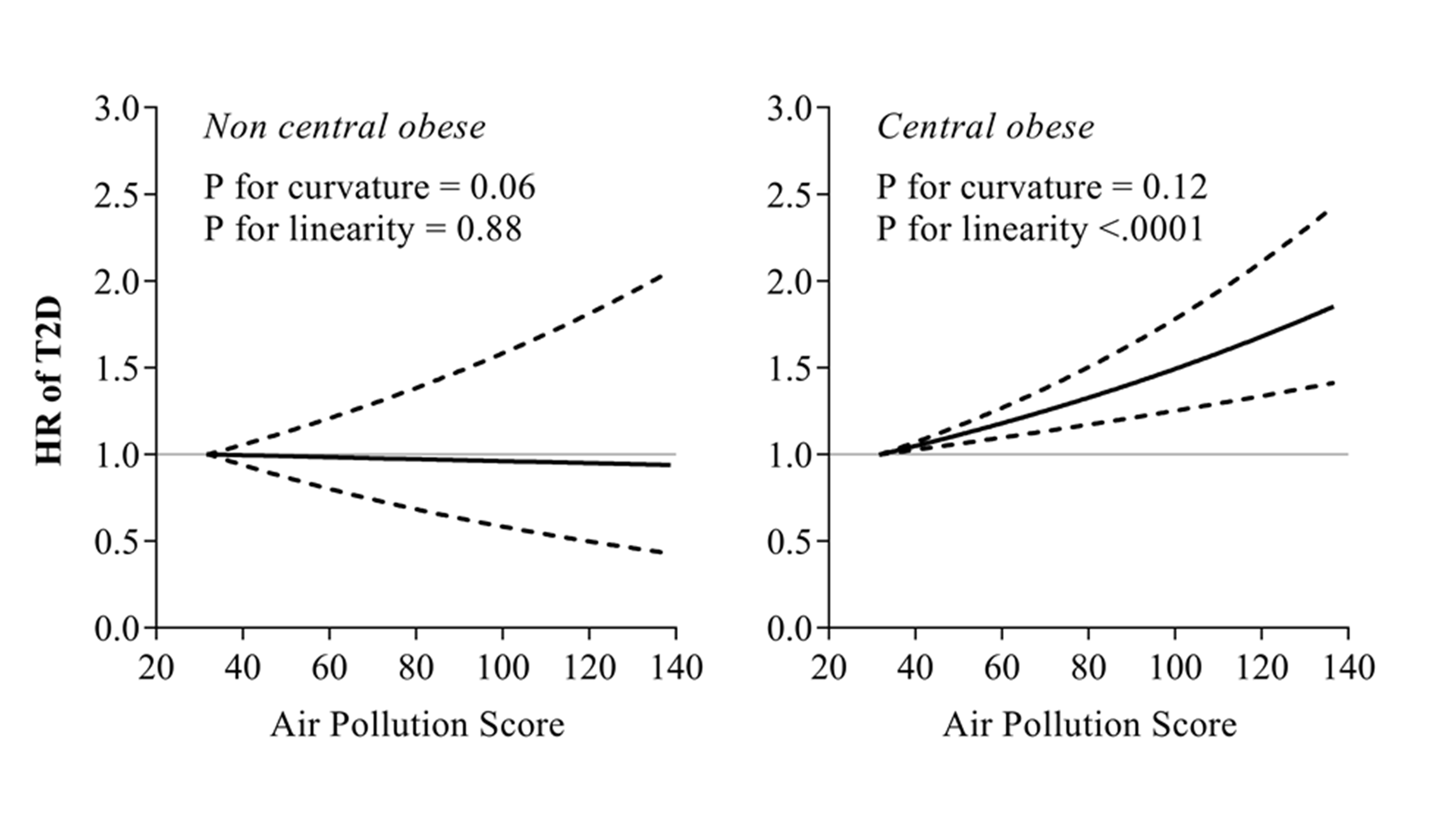

Supplement: S1 Fig — Dashed lines represent the 95% CIs of the HR. Multivariable models were adjusted for age, sex, Townsend deprivation index, center, alcohol intake, smoking status, physical activity, sedentary hours, healthy diet score, systolic blood pressure, antihypertension meds, high cholesterol, and T2D GRS. Sample size for noncentral obese and central obese subgroup were 177,791 and 271,215, respectively. CI, confidence interval; GRS, genetic risk score; HR, hazard ratio; T2D, type 2 diabetes. (TIF) [file pmed.1003767.s009.tif]

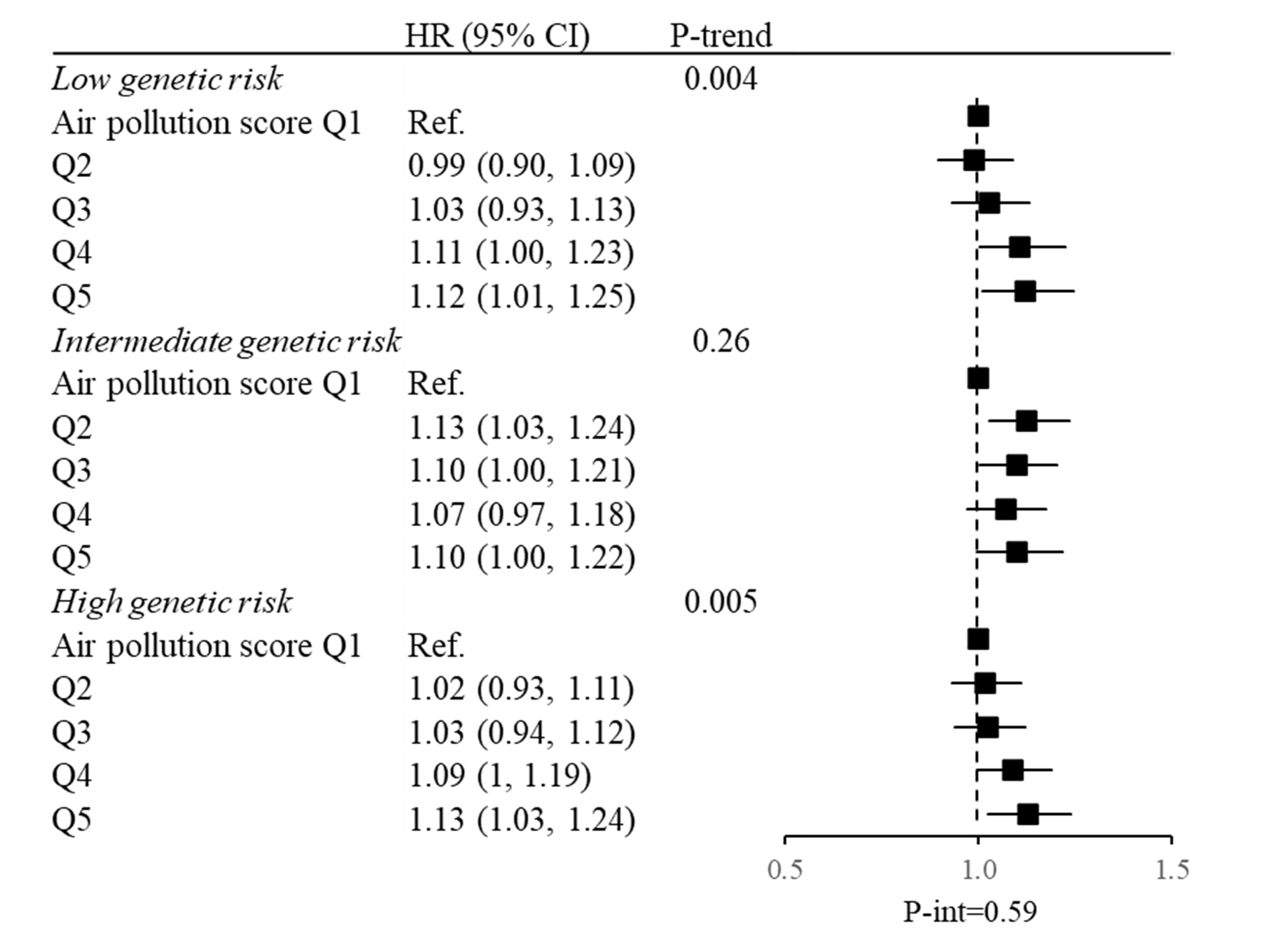

Supplement: S2 Fig — Stratified analyses were performed by tertiles of obesity GRS. Multivariable models were adjusted for age, sex, Townsend deprivation index, center, alcohol intake, smoking status, physical activity, sedentary hours, healthy diet score, BMI, systolic blood pressure, antihypertension meds, and high cholesterol. BMI, body mass index; CI, confidence interval; GRS, genetic risk score; HR, hazard ratio; T2D, type 2 diabetes. (TIF) [file pmed.1003767.s010.tif]

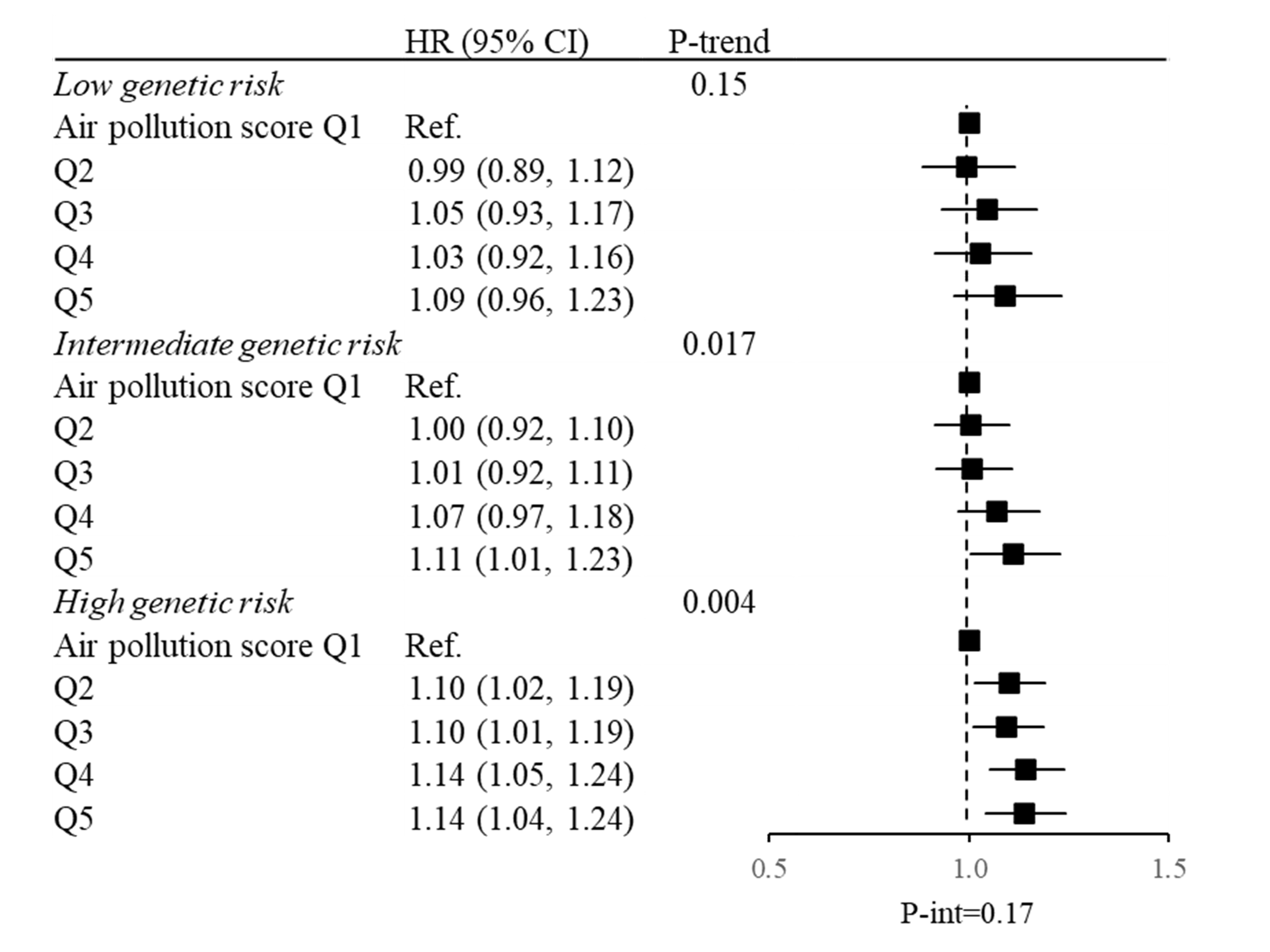

Supplement: S3 Fig — Stratified analyses were performed by tertiles of T2D GRS. Multivariable models were adjusted for age, sex, Townsend deprivation index, center, alcohol intake, smoking status, physical activity, sedentary hours, healthy diet score, BMI, systolic blood pressure, antihypertension meds, and high cholesterol. BMI, body mass index; CI, confidence interval; GRS, genetic risk score; HR, hazard ratio; T2D, type 2 diabetes. (TIF) [file pmed.1003767.s011.tif]
